# Acupuncture for fibromyalgia syndrome: an overview of systematic reviews and meta-analyses

**DOI:** 10.3389/fmed.2025.1712065

**Published:** 2025-12-05

**Authors:** Tae-Young Choi, Ji Hee Jun, Hye Won Lee, Myeong Soo Lee

**Affiliations:** 1 KM Science Research Division, Korea Institute of Oriental Medicine, Daejeon, Republic of Korea; 2 KM Convergence Research Division, Korea Institute of Oriental Medicine, Daejeon, Republic of Korea

**Keywords:** acupuncture therapy (AT), fibromyalgia syndrome (FMS), pain relief, GRADE assessment, systematic review, meta-analysis

## Abstract

**Objective:**

Numerous systematic reviews (SRs) and meta-analyses (MAs) have investigated the clinical effectiveness of acupuncture therapy (AT) for fibromyalgia syndrome (FMS); however, the quality and consistency of the evidence remain unclear. This study aimed to evaluate the methodological quality and strength of evidence in published SRs and MAs on the efficacy and safety of AT for FMS.

**Methods:**

A comprehensive literature search was conducted in 11 databases up to May 2025. Two reviewers independently selected studies, extracted data, and assessed methodological quality using A MeaSurement Tool to Assess systematic Reviews-2 (AMSTAR-2), reporting quality via Preferred Reporting Items for Systematic Reviews and Meta-Analyses (PRISMA) 2020 and certainty of evidence with Grading of Recommendations Assessment, Development, and Evaluation (GRADE).

**Results:**

Thirteen SRs and MAs were included. According to AMSTAR-2, only one SR and MA was rated high quality, three were moderate, and eight were low or critically low, respectively. Following the PRISMA guidelines, five SRs demonstrated compliance >85%. No high-quality evidence with GRADE assessment was found. The overall quality of evidence in the included SRs ranged from “very low” to “moderate.” AT was found to be superior to sham AT or standard pharmacological therapies (SPT) in treating FMS and pain. Evidence from 10 SRs (collectively reporting on 63 outcomes) indicated that AT provided superior pain relief than sham AT or SPT. AT was more effective than sham AT (Visual Analog Scale; mean difference [MD] −1.04 [−1.70, −0.38], *p* = 0.002) and SPT (MD −1.77 [−2.10, −1.44], *p* < 0.00001) in reducing pain. It also showed significantly better long-term pain relief (standard mean difference [SMD] 0.40 [−0.77, −0.03], *p* = 0.03), lower Fibromyalgia Impact Questionnaire scores (SMD −0.69 [−0.91, −0.47], *p* < 0.00001), reduced fatigue (SMD −0.03 [−0.42, 0.35], *p* = 0.87), reduced number of tender points (SMD −2.38 [−3.40, −1.37], *p* < 0.00001), and increased pain pressure threshold (SMD 0.31 [0.02, 0.61], *p* = 0.04). No adverse effects attributed to AT were reported.

**Conclusion:**

The current SRs and MAs provide low-quality evidence for the effectiveness of AT in treating FMS. Robust and well-designed studies using standardized methods are needed to provide more reliable and convincing evidence.

**Systematic review registration:**

https://www.crd.york.ac.uk/PROSPERO/view/CRD42024536968, Identifier CRD42024536968.

## Introduction

Fibromyalgia syndrome (FMS) is a complex chronic pain disorder predominantly affecting women, with a prevalence of 2–4% (1). The most common complaint is musculoskeletal discomfort, characterized by pain and stiffness in various areas, including the cervical spine, thoracic spine, shoulder girdle, and pelvic girdle. In addition, people with FMS often experience tiredness, changes in sleep patterns, and symptoms of depression and anxiety (2, 3). The exact cause of FMS is still unknown; however, some studies suggest that it may be associated with factors such as sensitization of the central nervous system, excessive activation of N-methyl-D-aspartate receptors, and inflammatory processes (4, 5). Diagnosis is primarily based on criteria such as those outlined by the American College of Rheumatology (ACR).

The current approach to treating FMS focuses on relieving symptoms such as pain and fatigue while also improving overall quality of life, including sleep and physical function. Primary treatment approaches include the use of anti-depressants, non-opioid analgesics, and opioid analgesics. Among these options, amitriptyline stands out as an effective drug recommended in the FMS treatment guidelines by American Pain Society (6). However, standard pharmacological therapies (e.g., gabapentinoids, tricyclic antidepressants; SPT) often have potential side effects, limiting the current medical treatment of FMS. These limitations and potential side effects create a substantial unmet need for effective and well-tolerated therapies. This situation underscores the importance of exploring and rigorously evaluating non-pharmacological treatments, such as AT, which can be an essential component of a comprehensive, patient-centered management strategy given the chronic nature of FMS. Therefore, non-pharmacological treatments such as education, exercise, and cognitive behavioral therapy are commonly used as initial treatment techniques (4, 5).

Acupuncture therapy (AT) is considered one of the important non-pharmacological treatments available for FMS. Extensive research on AT has produced clinical evidence supporting its efficacy in treating FMS. Similar to AT, systematic reviews (SRs) suggest Balneotherapy (BT) provides meaningful benefits for pain and quality of life in FMS (7, 8). However, the certainty of this evidence is consistently rated from Very Low to Moderate. This shared lack of high-certainty evidence highlights a pervasive methodological limitation across major non-pharmacological treatments for FMS. Although the exact mechanism of AT for FMS remains unclear, two main hypotheses have been proposed. The first hypothesis proposes that inserting needles into specific locations stimulates A-delta and C-afferent nerve fibers, which then transmit signals to various locations in the central nervous system, triggering the release of endogenous opioids. The second hypothesis suggests that AT triggers the release of neurotransmitters that downregulate the expression of A-delta fibers, resulting in long-term pain relief (4, 9).

Despite these promising findings, there remains a notable divergence in the interpretation of AT’s effectiveness. Numerous studies have shown that AT has therapeutic benefits and advantages in the management of chronic pain (10), insomnia (11), and improvement of anxiety and depression (12), particularly in chronic soft tissue pain problems (13). However, the European League Against Rheumatism (EULAR) recommendations for FMS therapy and management imply that there is insufficient evidence to conclusively endorse AT as a treatment (5). This contrast between clinical observations and formal guideline recommendations underscores a critical issue in the evaluation of AT research. It highlights the need for rigorous assessment of evidence quality to determine whether the perceived lack of support reflects true inefficacy or methodological limitations in existing studies.

A previously published comprehensive study providing an overview of complementary and alternative medicine (CAM) treatments for FMS demonstrated the effectiveness of AT in alleviating symptoms (14). Authors suggest that ‘existing overviews of FMS have limitations in accurately assessing the true effectiveness of acupuncture due to the lack of differentiation among the various types of CAM used.’ We confirm that previous overview studies primarily focused on broad CAM categories, and did not conduct dedicated, detailed sub-group analyses focusing specifically on the heterogeneity of acupuncture techniques or sham control variations, thereby limiting their clinical utility. Our study is thus essential to provide a focused and high-quality assessment.

Therefore, the main objective of this overview was to thoroughly analyze and summarize SRs that specifically focus on AT for FMS, offering an objective and comprehensive evaluation of its efficacy and safety. Furthermore, this overview summarizes the outcome indicators used in meta-analyses (MAs), providing a valuable resource for future clinical research.

## Methods

### Reporting guidelines and registration compliance

This review was documented following the Preferred Reporting Items for Overview of Systematic Reviews (PRIOR) guidelines (15). The protocol has been registered on PROSPERO (CRD42024536968).

### Literature search strategy

The time frame for retrieving information ranged from the establishment of the database until May 2025. The search included 11 databases. Three international databases (PubMed, EMBASE, and the Cochrane Library), two Chinese databases (China National Knowledge Infrastructure and Wanfang), and six Korean databases (KoreaMed, Oriental Medicine Advanced Search Integrated System, DBpia, Korean Medical Database, Research Information Service System, and Korean Studies Information Services System) were searched. The search terms using the Medical Subject Heading, consisted of (“fibromyalgia” OR “fibromyalgia syndrome”) AND (“acupuncture” OR “electro-acupuncture”) AND (“systematic review” OR “meta-analysis”) in English, Chinese, and Korean (Supplementary material 1). Additional relevant literature was manually searched for references.

### Eligibility criteria for included studies

#### Inclusion criteria

1. Randomized controlled trials (RCTs) served as the basis for SRs, with a minority of quasi-RCTs included and evaluated alongside RCTs. SRs were considered for inclusion regardless of whether they contained a meta-analysis, and there were no restrictions on language inclusion.
2. FMS diagnosis was established definitively through the use of criteria such as those outlined by the ACR. This diagnosis was independent of factors such as type, sex, age, and disease progression.
3. The treatment group received AT or a combination of AT and control group. The control group received placebo, sham AT, no treatment, or conventional medication.
4. The primary outcome measures focused on the effectiveness of validated pain assessment tools. These measures included the count tender point upon palpation, pain intensity, or standardized pain scales such as the Visual Analog Scale (VAS), McGill Pain Questionnaire (MPQ), and Chronic Pain Grade Scale.

#### Exclusion criteria

1. Duplicate articles.
2. Contained incomplete data [defined as reviews where the quantitative results of primary outcomes (e.g., mean, standard deviation, or effect size)] were not fully reported or could not be reliably extracted for summarizing the evidence.
3. Narrative reviews, overviews, and network meta-analysis.
4. The research object is FMS and FMS combined with other diseases.
5. Studies where it was too difficult to isolate the individual effects of AT and other traditional medicine combined approaches.

### Study selection and data extraction process

Literature was screened and extracted independently by two researchers (T-YC and JHJ) and cross-checked. If there was any disagreement, it was discussed and negotiated, or a third expert (MSL) adjudicated. The extracted literature information included authors, publication year, sample size, intervention measures, results, quality assessment tools, and main conclusions.

### Assessment of study overlap among SRs

The extent of overlap among primary studies included in SRs was evaluated by constructing citation matrices for the SRs. We employed the “corrected covered area” (CCA) index to quantify the degree of overlap, as previously described (16, 17). The CCA is calculated using the formula CCA = (*N* – *r*)/(rc – r), where *N* represents the total number of included publications across all reviews (i.e., the sum of all marked cells in the citation matrix), *r* denotes the number of unique primary studies (rows), and c indicates the number of SRs (columns). The CCA reflects the proportion of repeated occurrences of primary studies relative to the maximum possible overlap, adjusted for the number of unique studies. According to established thresholds, a CCA value below 5 indicates “slight overlap,” 6–10 denotes “moderate overlap,” 11–15 suggests “high overlap,” and values of 15 or greater represent “very high overlap.”

### Quality assessment methodology

The methodological quality of the SRs and MAs was assessed using the AMSTAR-2 scale (18). This scale contains 16 items that can be answered as “Yes, Partially yes, or No.” If two or more of the key questions (2, 4, 7, 9, 11, 13, 15) are omitted, the quality is considered “Critically low.” If one key item is omitted, the quality is considered “Low.” If two or more of the non-critical items, excluding the important ones mentioned above, are omitted, the quality is considered “Moderate.” Additionally, if important items and one missing or unimportant item are omitted, the quality is rated as “High.”

The reporting quality of the SR/MA was assessed using the PRISMA 2020 guidelines (19). These guidelines includes 27 elements that can be answered as “Yes, Partially yes, or No.” The results were reported as a ratio.

The Grading of Recommendations Assessment, Development, and Evaluation (GRADE) approach (20) assesses the Certainty of evidence (CoE) for each outcome. The CoE is categorized as High, Moderate, Low, or Very low. Evidence is graded as high based on RCTs; however, it may be downgraded based on various factors such as risk of bias (RoB), indirectness of evidence, unexplained heterogeneity or inconsistency of results, imprecision of results, and publication bias.

## Results

### Literature search

**Figure 1:**
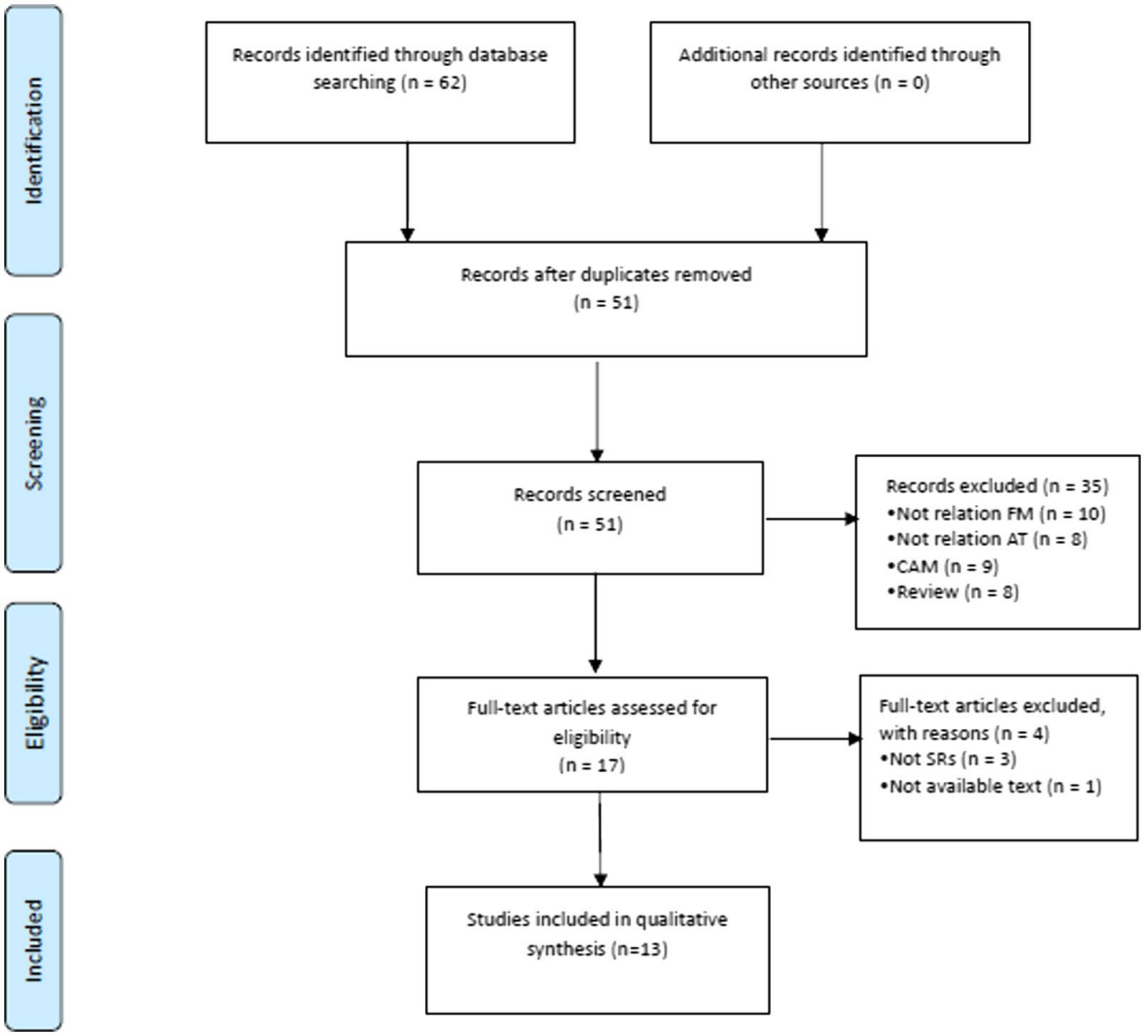
Flow chart of study selection. AT, acupuncture therapy; CAM, complementary and alternative medicine; SRs, systematic reviews.

According to the search strategy, 62 eligible studies were identified, and 11 duplicated studies were deleted. Thirty-five studies were excluded based on the title or the abstract being irrelevant to the topic. After screening through the full-text reviews, 13 SRs (21–33) were finally selected for analysis (Figure 1).

### Characteristics of SRs

Thirteen SRs and MAs regarding AT for FMS were published between 2007 and 2022. Among these, six SRs originated from China (25, 26, 28, 30, 32, 33), two SRs from UK (21, 22), two SRs from Spain (23), one SR from Germany (24), one SR from Korea (29), and one SR from Australia (Table 1) (27). Overall, 11 SRs were published in English (21–24, 26–32) and two were in Chinese (25, 33).

**Table 1:** Characteristics of included SRs/MAs of AT for FMS.

First author (year) country (ref)	Search date diagnosis No. of primary studies (sample size)	Intervention	Comparator	· Outcome ***·*** Quality assessment tools ***·*** Adverse events mentioned	Conclusion (quote from the original paper)	Protocol
Mayhew (2007) UK (21)	**·** NR 2006 **·** ACR **·** 5 (316)	AT	Sham AT SPT	***·*** Pain ***·*** Jadad score ***·*** Yes (no serious adverse events)	… not supported by the results from rigorous clinical trials. …, AT cannot be recommended for FMS.	No
Daya (2007) UK (22)	**·** June 2006 **·** ACR **·** 4 (158)	AT	Sham AT	**·** Pain **·** van Tulder **·** Yes (bruising/soreness)	No recommendation can be given regarding the prescription of AT for FMS.	No
Martin-Sanchez (2009) Spain (23)	**·** January 2008 **·** ACR **·** 6 (323)	AT	Sham AT	**·** Pain (VAS) **·** NR **·** NR	… no evidence of benefit resulting …	No
Langhorst (2010) Germany (24)	**·** July 2009 **·** ACR **·** 7 (385)	AT	Sham AT Simulated AT	**·** Pain/fatigue/sleep/Physical function **·** Cochrane RoB/van Tulder score **·** Yes (not details)	AT cannot be recommended for the management of FMS.	No
Gao (2010) China (25)	**·** NR 2009 **·** ACR **·** 5 (306)	AT	SPT	**·** ER **·** Cochrane RoB/Jadad score **·** NR	…efficacy of AT for the treatment of FMS.	No
Cao (2013) China (26)	**·** January 2012 **·** ACR/IASP **·** 16 (1081)	AT	Sham AT SPT	**·** Changes of pain (VAS)/pain (VAS)/No. of tender points/depression/sleep/FQI **·** Cochrane RoB **·** Yes (bruising/nausea/fainting/discomfort of needle insertion/simulated needle insertion/bleeding)	Acupoint stimulation appears to be effective in treating FMS compared with SPT.	No
Deare (2013) Australia (27)	**·** January 2012 **·** ACR **·** 9 (395)	AT	Sham AT SPT	**·** Pain/physical function (SF-36)/wellbeing/sleep/fatigue/stiffness **·** Cochrane RoB **·** Yes (not details)	FMS may consider using AT alone or with exercise and SPT.	Cochrane protocol
Bai (2014) China (28)	**·** March 2012 **·** ACR **·** 9 (592)	AT	Sham AT SPT	**·** Pain (NRS, VAS)/PPT/ER **·** Cochrane RoB **·** Yes (bruising/soreness/vasovagal symptoms/palpitation/mouth dryness/dizziness/perspiration/constipation)	…there was not enough evidence to prove the efficacy of AT for the treatment of FMS.	No
Kim (2019) Korea (29)	**·** August 2018 **·** ACR **·** 10 (690)	AT	Sham AT	**·** Pain/general status **·** Cochrane RoB · Yes (local edema/bruising/soreness/worsening symptoms/tiredness/headache/vasovagal symptoms)	AT is more effective than sham AT for …reforming general status in FMS posttreatment. …reduces fatigue was not found.	PROSPERO CRD42018076279
Zhang (2019) China (30)	**·** May 2018 **·** ACR **·** 12 (824)	AT	Sham AT SPT	**·** Pain VAS/pain SF-MPQ/FIQ **·** Cochrane RoB **·** Yes (bruising/soreness/nausea/discomfort of needle insertion/aggravation of symptoms)	AT is an effective and safe treatment for patients with FMS, and this treatment can be recommended for the management of FMS.	PROSPERO CRD42018094636
Isabel (2020) Spain (31)	**·**December 2018 **·** ACR **·** 13 (789)	AT (dry needling)	Sham AT SPT UC	**·** Pain/FIQ/PPT/Quality of life (SF-36) **·** Cochrane RoB **·** NR	… considered effective for pain relief, as well as for producing a short-term increase in the pain pressure threshold, an improvement in quality of life…	PROSPERO CRD42019128750
Zheng (2022) China (32)	**·**September 2021 **·** ACR **·** 13 (923)	AT/EA	Sham AT	**·** Pain/fatigue/sleep/physical function/wellbeing **·** Cochrane RoB **·** Yes (bruising/nausea/fainting/discomfort of needle insertion)	Moderate quality of evidence supports AT in reducing pain in patients with FMS. AT is recommended as a treatment for FMS.	PROSPERO CRD42021267743
Yang (2022) China (33)	**·**December 2021 **·** ACR **·** 14 (776)	AT	SPT	**·** ER/FIQ/pain VAS/number of tender points/AIS **·** Cochrane RoB **·** NR	AT in the treatment of FMS has better short-term analgesic efficacy and fewer adverse events than SPT.	No

ACR, American College of Rheumatology; AIS, Athens Insomnia Scale; AT, acupuncture therapy; ER, efficacy rate; FIQ, fibromyalgia impact questionnaire; FMS, fibromyalgia syndrome; IASP, International Association for the Study of Pain; NR, not reported; NRS, numerical rating scale; PPT, pain pressure threshold; QOL, quality of life; RoB, risk of bias; VAS, visual analogue scale; SF-MPQ, short form of McGill Pain Questionnaire; SPT, standard pharmacological therapies.

Twelve of the SRs included between 4 and 16 RCTs, with participant numbers ranging from 158 to 1,081. In all SRs, FMS was diagnosed using the ACR criteria. In only one SR, both the ACR and International Association for the Study of Pain (IASP) criteria were used together (26). The interventions in the therapy group were mainly AT. Only one SR included AT and dry needling, which are invasive techniques (31). The control group mainly consisted of conventional medicine and sham AT.

Most SRs used the Cochrane RoB tool for assessing the methodological quality (24–33). The Jadad (21, 25) and van Tulder score (22, 24) were each used in two SRs. Only one SR quality assessment tool was not mentioned (23). Meta-analysis was performed in 10 SRs (23–33), and only one SR was a Cochrane review (26). Four SRs had their protocol registered in PROSPERO (29–32).

### Overlap

These 13 SRs included a total of 45 primary studies, which were published between 1989 and 2021. All of these trials evaluated pain in FMS. The most frequently included primary studies were Martin 2006 (*n* = 11) (34), Assefi 2005 (*n* = 10) (35), Deluze 1992 (*n* = 9) (36), and Harris 2005 (*n* = 9) (37).

A total of 13 SRs was included in this review. N indicates 127, r indicates 45, and c indicates 13. The formula CCA = (127–45)/(45 × 13–45) = 0.15 indicates very high overlap. The overlap matrix is shown in Supplementary material 2.

### AMSTAR-2

**Figure 2:**
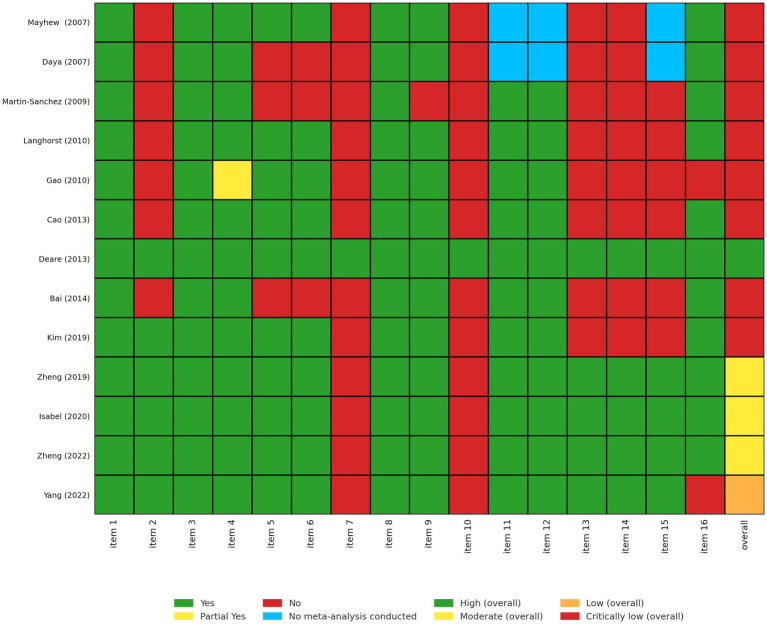
Assessment of all included systematic reviews using AMSTAR-2. Each systematic review was evaluated across 16 domains of the AMSTAR-2 tool (1): inclusion of PICO components in research questions and criteria (2); prior establishment and reporting of review methods with protocol deviations justified (3); explanation of chosen study designs (4); use of a comprehensive literature search strategy (5); duplicate study selection (6); duplicate data extraction (7); provision and justification of excluded studies (8); detailed description of included studies (9); appropriate risk of bias (RoB) assessment methods (10); reporting of funding sources for included studies (11); use of appropriate statistical methods in meta-analyses (12); assessment of RoB impact on meta-analysis results (13); consideration of RoB in result interpretation (14); explanation of heterogeneity (15); investigation and discussion of publication bias; and (16) disclosure of potential conflicts of interest and funding. Responses were coded as Yes (Y), Partial Yes (PY), No (N), or No Meta-Analysis conducted (NMA).

Among the 13 SRs, one was of high quality, three were of moderate quality, one was of low quality, and eight were of critically low quality, according to the AMSTAR-2 (Figure 2). All of the SRs provided a detailed explanation of PICO, study design selection, and comprehensive search strategies and included studies (Items 1, 3, 4, and 8). Our findings showed that the main weaknesses were: (1) lack of protocol registration (Item 2); (2) not providing a complete list of excluded studies and justifying the exclusions (Item 7); (3) not reporting the sources of funding for the studies that were included in the SRs (Item 10), and (4) not considering the RoB assessment when interpreting the results of the review (Item 13).

### PRISMA 2020

**Figure 3:**
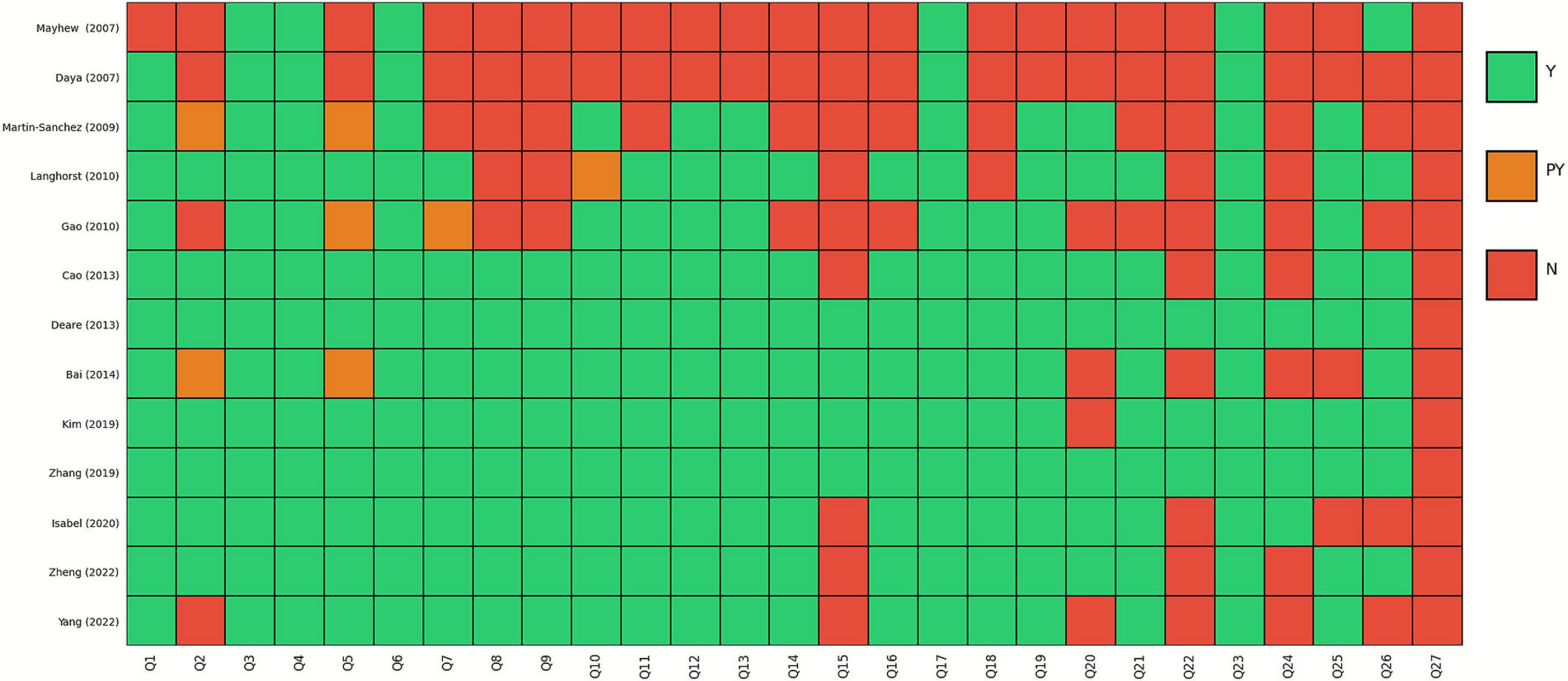
Assessment of all included systematic reviews using PRISMA checklist. Each systematic review was evaluated against the following PRISMA checklist items: Q1. Title; Q2. Abstract; Q3. Rationale; Q4. Objectives; Q5. Eligibility criteria; Q6. Information sources; Q7. Search strategy; Q8. Selection process; Q9. Data collection process; Q10. Data items; Q11. Study risk of bias assessment; Q12. Effect measures; Q13. Synthesis methods; Q14. Reporting bias assessment; Q15. Certainty assessment; Q16. Study selection; Q17. Study characteristics; Q18. Risk of bias in studies; Q19. Results of individual studies; Q20. Results of syntheses; Q21. Reporting biases; Q22. Certainty of evidence; Q23. Discussion; Q24. Registration and protocol; Q25. Support; Q27. Availability of data, code, and other materials.

We used the PRISMA checklist to evaluate the quality of reporting in the included SRs. Item 3 (rationale), Item 4 (objects), Item 6 (information sources), Item 17 (study characteristics), and Item 23 (discussion) were reported adequately, scoring 100%. However, Item 15 (describe any methods used to assess certainty or confidence), Item 22 (CoE), Item 24 (registration and protocol), and Item 27 (availability of data, code, and other materials) were insufficiently described. Overall, five SRs (26, 27, 29, 30, 32) exhibited over 85% compliance (Figure 3).

### Effectiveness of AT for FMS

Thirteen SRs have summarized the evidence on the effectiveness of AT in treating FMS, and out of those, 10 SRs have conducted MAs. The summary of outcomes from these SRs is presented in Tables 1, 2. The majority of studies indicated that AT has beneficial effects on managing patients’ conditions and that it shows promise as an intervention for symptom management and improving the quality of life for patients with FMS. The evidence from 10 SRs, consisting of 63 outcomes, indicates that AT provides better pain relief than the control interventions (sham AT or SPT).

**Table 2:** Quality of evidence in the included SRs assessed by the GRADE approach.

First author (year) (ref)	Intervention vs. comparator	Outcome	No. of studies (no. of patients)	Relative absolute (95% CI)	*p*-value	Quality of evidence
Martin-Sanchez (2009) (23)	AT vs. Sham AT	Pain (VAS)	4 (257)	SMD 0.02 [−0.24, 0.28]	NS	Moderate
Langhorst (2010) (24)	AT vs. Sham AT	Pain	7 (242)	SMD -0.25 [−0.49, −0.02]	*=0.04*	Moderate
		Fatigue	3 (147)	SMD 0.04 [−0.32, 0.39]	NS	Very low
		Sleep	2 (87)	SMD 0.05 [−0.79, 0.83]	NS	Very low
		Physical function (SF-36)	3 (149)	SMD -0.15 [−0.61, 0.91]	NS	Very low
		Pain (F/U)	2 (87)	SMD -0.11 [−0.72, 0.49]	NS	Very low
		Physical function (F/U)	2 (87)	SMD -0.05 [−0.47, 0.37]	NS	Very low
Gao (2010) (25)	AT vs. SPT	ER	5 (190)	RR 1.49 [1.20, 1.73]	*<0.00001*	Very low
Cao (2013) (26)	AT vs. Sham AT	Changes of pain VAS	6 (330)	SMD -0.09 [−0.32, 0.14]	NS	Moderate
		Pain (VAS)	6 (360)	SMD -0.22 [−0.51, 0.07]	NS	Moderate
		Depression	1 (50)	MD -0.33[−0.90, 023]	NS	Very low
		FIQ	1 (50)	MD -4.30 [−11.08, 2.48]	NS	Very low
		Fatigue	2 (164)	SMD -0.05 [−0.41, 0.30]	NS	Very low
	AT vs. SPT	Pain (VAS)	5 (285)	SMD -0.74 [−1.13, −0.35]	*=0.0002*	Moderate
		No. of tender points	3 (195)	SMD -2.38 [−3.40, −1.37]	*<0.00001*	Very low
		Depression	2 (90)	SMD -0.67 [−1.10, −0.25]	*=0.02*	Very low
		Sleep	3 (195)	SMD -0.32 [−0.63, −0.01]	*=0.04*	Very low
		FIQ	1 (30)	MD -4.60 [−12.42. 3.22]	NS	Very low
		Fatigue	1 (30)	MD -0.27 [−0.99, 0.45]	NS	Very low
Deare (2013) (27)	AT vs. Sham AT	Pain	6 (286)	SMD -0.14 [−0.53, 0.25]	NS	Moderate
		Physical function (SF-36)	1 (56)	MD -5.80 [−10.91, −0.69]	*=0.03*	Very low
		Well-being	3 (200)	SMD 0.29 [−0.44, 1.01]	NS	Low
		Sleep	3 (200)	SMD 0.16 [−0.29, 0.61]	NS	Moderate
		Fatigue	3 (200)	SMD -0.10 [−0.81, 0.61]	NS	Low
		Stiffness	2 (104)	SMD -0.45 [−0.84, −0.06]	*=0.02*	Very low
		Pain (F/U)	2 (145)	SMD -0.12 [−0.52, 0.28]	NS	Very low
		Well-being (F/U)	2 (104)	SMD -0.03 [−0.87, 0.81]	NS	Very low
		Sleep (F/U)	2 (104)	SMD -0.09 [−0.44, 0.26]	NS	Very low
		Fatigue (F/U)	2 (154)	SMD 0.04 [−0.52, 0.59]	NS	Very low
		Stiffness (F/U)	1 (49)	MD -0.30 [−1.60, 1.00]	NS	Very low
Bai (2014) (28)	AT vs. Sham AT	Pain (NRS)	2 (105)	MD -1.06 [−10.41, 8.30]	NS	Very low
	AT vs. SPT	Pain (VAS)	1 (56)	MD -2.27 [−3.05, −1.49]	*<0.00001*	Very low
		ER	1 (36)	RR 1.38 [1.00, 1.91]	*=0.05*	Very low
		PPT	1 (58)	MD 0.69 [0.38, 1.00]	*<0.00001*	Very low
Kim (2019) (29)	AT vs. Sham AT	Pain	11 (559)	SMD -0.49 [−0.79, −0.20]	*=0.001*	Moderate
		FIQ	6 (389)	SMD -0.69 [−0.91, −0.47]	*<0.00001*	Moderate
Zhang (2019) (30)	AT vs. Sham AT	Pain (VAS)	9 (528)	MD -1.04 [−1.70, −0.38]	*=0.002*	Low
		Pain (SF-MPQ)	2 (70)	MD -1.23 [−4.74, 2.27]	NS	Low
		FIQ	5 (307)	MD -13.39 [−21.69, −5.10]	*=0.002*	Moderate
		Pain (F/U)	3 (251)	MD -1.58 [−2.72, −0.44]	*=0.006*	Moderate
		FIQ (F/U)	3 (251)	MD -12.92 [−24.92, −0.93]	*=0.03*	Low
	AT vs. SPT	Pain (VAS)	2 (98)	MD -1.81 [−2.43, −1.18]	*<0.00001*	Very low
		Pain (VAS) (F/U)	1 (60)	MD -2.11 [−2.97, −1.25]	*<0.00001*	Very low
Isabel (2020) (31)	AT vs. Control (Sham AT, UC)	Pain	7 (372)	SMD -0.94 [− 1.44, −0.44]	*=0.0002*	Low
		FIQ	5 (310)	SMD -0.99 [−1.69, 0.29]	*=0.006*	Low
		PPT	2 (184)	SMD 0.31 [0.02, 0.61]	*=0.04*	Very low
		QOL (SF-36)	3 (211)	SMD 0.84 [0.30, 1.38]	*=0.002*	Moderate
Zheng (2022) (32)	AT vs. Sham AT	Pain	12 (715)	SMD -0.42 [−0.66, −0.17]	*=0.0009*	Moderate
		Fatigue	4 (251)	SMD -0.03 [−0.42, 0.35]	NS	Moderate
		Sleep	2 (151)	SMD -0.38 [−0.78, 0.02]	NS	Very low
		Physical function (SF-36)	3 (268)	SMD 0.14 [−0.51, 0.79]	NS	Low
		Stiffness	2 (104)	SMD -0.38 [−0.77, 0.01]	NS	Very low
		Well-being	4 (357)	SMD -0.86 [−1.49, −0.24]	=0.007	Low
		Pain (F/U)	4 (356)	SMD -0.40 [−0.77, −0.03]	*=0.03*	Moderate
		Fatigue (F/U)	2 (145)	SMD 0.04 [−0.52, 0.59]	NS	Low
		Sleep (F/U)	2 (98)	SMD -0.64 [−1.77, 0.49]	NS	Very low
		Physical function (F/U)	2 (98)	SMD -0.25 [−0.91, 0.41]	NS	Very low
		Well-being (F/U)	3 (307)	SMD -0.58 [−0.82, −0.35]	*<0.00001*	Moderate
Yang (2022) (33)	AT vs. SPT	ER	10 (543)	RR 1.25 [1.14, 1.37]	*<0.00001*	Low
		FIQ	3 (194)	−12.06 [−20.56, −3.57]	*=0.005*	Very low
		Pain (VAS)	7 (352)	−1.77 [−2.10, −1.44]	*<0.00001*	Low
		Number of tender points	5 (232)	−2.74 [−3.62, −1.87]	*<0.00001*	Very low
		AIS	2 (145)	−0.16 [−2.42, 2.10]	NS	Very low

AT, acupuncture therapy; CI, confidence interval; ER, efficacy rate; FIQ, fibromyalgia impact questionnaire; NRS, numerical rating scale; SPT, standard pharmacological therapies; PPT, pain pressure threshold; VAS, visual analogue scale; QOL, quality of life; RR, risk ratio; SMD, standardized mean difference; WMD, weighted mean difference; UC, usual care. The italicized values in Table 2 indicate statistically significant.

Specifically, AT was more effective than sham AT (mean difference [MD] -1.04 [−1.70, −0.38], *p* = 0.002) (30) and SPT (MD −1.77 [−2.10, −1.44], *p* < 0.00001) (33) in reducing pain (VAS). AT also showed better long-term pain relief for FMS (standard mean difference [SMD] 0.40 [−0.77, −0.03], *p* = 0.03) (32). Furthermore, AT was found to be more effective in reducing Fibromyalgia Impact Questionnaire scores (FIQ; SMD −0.69 [−0.91, −0.47], *p* < 0.00001) (29) and fatigue (SMD −0.03 [−0.42, 0.35], *p* = 0.87) (32). The number of tender points (SMD −2.38 [−3.40, −1.37], *p* < 0.00001) (26) and pain pressure threshold (PPT) (SMD 0.31 [0.02, 0.61], *p* = 0.04) (31) were also significantly reduced.

Assessments for core domains of FMS yielded a total of 47 outcomes (Supplementary material 2). The most commonly reported outcomes across studies were related to pain intensity, predominantly assessed using various pain indices (reported in five studies), VAS (six studies), and NRS (one study) scales. Other tools used to measure pain outcomes include PPT, efficacy rate, the short-form MPQ, changes in pain VAS, and the number of tender points. In addition to pain outcomes, fatigue (*n* = 5), sleep (*n* = 4), depression (*n* = 2), and stiffness (*n* = 2) were evaluated as comorbidities. Furthermore, the FIQ (*n* = 6), physical function (SF-36; *n* = 3), quality of life (SF-36; *n* = 1), and wellbeing (*n* = 2) were assessed (Supplementary material 3).

### Certainty of evidence (CoE)

Sixty-three outcomes extracted from the included SRs and MAs were evaluated using the GRADE approach to determine the CoE. A summary of the findings, including outcomes with moderate or very low CoE, is shown in Table 2. Specifically, no high CoE was found; 16 outcomes showed moderate, 12 outcomes showed low, and 35 outcomes showed very low CoE.

The RoB, inconsistency, and imprecision were the main reasons for downgrading. Most primary RCTs included in the SRs and MAs did not report allocation concealment, blinding, or selective reporting. Heterogeneity within studies downgraded the inconsistency. Imprecision was downgraded owing to the wide confidence intervals or small number of participants (<200). The details of the GRADE assessment are provided in Table 2 and Supplementary material 4.

### Adverse events (AEs)

Of all the 13 SRs, nine (21, 22, 24, 26–30, 32) mentioned the AEs of AT in the treatment of FMS. Three SRs (23, 25, 31, 33) did not describe any information on AEs, and six SRs (22, 26, 28–30, 32) reported AEs that were attributable to AT. A wide range of minor AEs was reported, which included bruising, soreness, discomfort of needle insertion, nausea, vasovagal symptoms, and fainting (Supplementary material 5). No serious AEs requiring medical management were reported.

## Discussion

### Summary of main findings

Although 13 SRs and MAs were included, a number generally considered sufficient for an overview of reviews, we must acknowledge this sample size as a limitation in generalizing our findings to the entire body of evidence for AT in FMS. Accordingly, our conclusion emphasizes the low quality of the existing evidence rather than making definitive statements about the absolute clinical effectiveness of AT.

To the best of our knowledge, this is the first comprehensive review of AT for the treatment of FMS. This review is also the first to examine the overall quality of the 13 SRs/MAs from 45 primary studies published between 2007 and 2022 and provide a summary of AT for FMS to provide a reference for clinical decision-making. The primary outcome measure showed that AT reduced pain and fatigue compared with sham AT or SPT, making it an acceptable and promising treatment option for FMS. However, primary results are still missing, with most studies having a low or critically low RoB and reporting quality. Therefore, high-quality RCTs and SRs and MAs are still needed to determine the effectiveness of AT in the treatment of f FMS. Caution is warranted when interpreting these findings as a result of the potential RoB in the evidence. AT may be considered a safe treatment modality for individuals with f FMS, given the absence of any major adverse reactions associated with its use. Mild adverse reactions such as bleeding or bruising at the sites of needling, pain, swelling, and pruritus may occur; however, these can generally be prevented by a proficient acupuncturist.

The fact that nearly half of the SRs originated from China suggests a high level of research activity in AT for FMS within that region. However, this geographical concentration may limit the generalizability of the findings to other populations or healthcare systems due to differences in clinical practices, cultural contexts, or research priorities that possibly influence study design, execution, and reported outcomes. Therefore, while a substantial body of evidence exists, its global applicability requires careful consideration, and contributions from more diverse geographical regions would benefit broader clinical recommendations.

Adhering to the PRIOR guidelines and registering the study protocol on PROSPERO clearly demonstrates this review’s commitment to methodological rigor and transparency. This approach contrasts with the lack of protocol registration observed in many included SRs. By adopting these high standards, this review enhances the trustworthiness and utility of its findings while simultaneously setting a higher benchmark for evidence synthesis in this field. This is essential for increasing the credibility of the results.

The primary reasons for downgrading the CoE in the GRADE assessment (RoB, inconsistency, and imprecision) are direct consequences of the systematic methodological weaknesses identified in the AMSTAR-2 and PRISMA assessments. For example, the lack of reporting on allocation concealment, blinding, or selective reporting in most primary RCTs directly increases the RoB. Heterogeneity within studies often stems from variations in interventions, patient populations, or outcome measures, leading to inconsistent findings. The observed high heterogeneity and consistently low evidence certainty (GRADE) for acupuncture may largely be attributed to methodological inconsistencies among the included primary studies. Notably, the control interventions described as ‘sham acupuncture’ in the systematic reviews were not uniform, involving variations such as the use of non-penetrating sham needles (Park-Sham device), shallow insertion at non-acupuncture points, or minimal electrical stimulation. This lack of standardized sham procedures significantly contributes to the performance bias and heterogeneity within the evidence base.

### Limitations and future directions

Our review identified several limitations inherent in the existing body of evidence. First, potential publication bias cannot be excluded, as studies with small sample sizes often report positive effects, which may lead to the overrepresentation of favorable findings in published literature. Second, significant heterogeneity was evident across the included reviews. This heterogeneity stems from variations not only in the control interventions (sham type) and acupuncture techniques (manual, electroacupuncture, point selection) but also in the geographical regions and diagnostic criteria used in the primary studies. These factors collectively contribute to the low confidence in the estimated effects, as reflected by the low GRADE ratings. Furthermore, the small sample sizes and wide confidence intervals of the included studies contribute to imprecision. These issues are not isolated but interconnected problems that systematically undermine the trustworthiness of the accumulated evidence. To significantly improve the CoE in this field, future research must actively address these fundamental methodological shortcomings through rigorous study design, appropriate blinding strategies (even for non-pharmacological interventions), adequately powered studies, and transparent reporting of all methodological details.

### Implications for further study

We conducted a thorough evaluation of the published SRs using assessment tools such as AMSTAR-2, PRISMA, and GRADE. In the AMSTAR-2 evaluation, overall confidence in the outcomes was found to be critically low. This finding was due to several factors, including the lack of registration and funding data, incomplete search strategy, absence of a list and justification for excluded articles, and insufficient explanation of the RoB. Future research should aim to address these shortcomings. All the studies included in this analysis had an average reporting quality based on the PRISMA 2020 checklist. Specifically, all six elements (Items 1, 2, 4, 8, 19, 20, and 21) were complete. However, only two SRs achieved a compliance rate of 85.2% (34) and 100% (12). Most of the SRs analyzed focused on FMS and were conducted and published in China. In future studies, it is recommended to use the Consolidated Standards of Reporting Trials (38) and PRISMA checklists (39) to enhance the reporting quality of SRs and MAs, thus reducing potential selection bias. The quality of evidence for the included SRs was assessed using the GRADE approach. For most outcomes, the strength of the evidence was rated as moderate, low, or very low. Many outcome measures were downgraded due to bias from randomization, distribution concealment, or lack of blinding in experiments. Given the unique nature of AT, blinding patients was not feasible.

Efforts have been made to establish standard diagnostic criteria for FMS, such as the 2016 ACR criteria (40). These criteria require a combination of widespread pain, tenderness, and other symptoms. However, they are not universally accepted and have limitations, including a lack of specificity and reliance on subjective symptoms. Further research is necessary to refine and validate diagnostic criteria for FMS. Similarly, there is a need for standardized symptom evaluation tools for FMS. Various instruments, such as the FIQ, SF-36, PSQI, MFI, and HAQ, have been developed to assess pain, fatigue, sleep, and other symptoms of FMS. However, the choice of instruments may vary depending on the specific symptoms and treatment goals. In addition, there is a lack of consensus on the instruments to be used and interpretation of the results. These measures may not capture the full range of symptoms and functional impairments in FMS. Therefore, more comprehensive and patient-centered outcome measures are needed. Standardized diagnostic criteria, symptom evaluation tools, and outcome measures can enhance the consistency and comparability of clinical practice and research, ultimately improving the outcomes and quality of life for people with FMS.

### Limitations

This review has a few limitations that should be considered. First, the data we reviewed only included papers written in English, and Chinese; therefore, there may be gaps in the research. AT research is conducted worldwide; therefore, studies in other languages may have been missed, resulting in a bias in the selection of studies. This bias could also lead to a lower number of studies included in our analysis. Second, some overlap may exist among the primary studies included in the SRs we examined; however, this has not been assessed thoroughly. The overlap can result in the misreporting of data, such as participant numbers and primary studies, leading to “double counting” of data in the reported MAs. Third, several relevant RCTs conducted prior to the publication of included reviews were not captured. This may be attributable to limitations in the search strategies, restricted databases used, differences in eligibility criteria, or methodological shortcomings identified in AMSTAR2 assessments. These factors highlight the need for comprehensive, regularly updated systematic review to ensure evidence completeness. Finally, the assessment of methodological quality and evidence quality using AMSTAR-2, PRISMA, and GRADE is a subjective process. Different reviewers may have diverse views on the various influencing factors, which could introduce bias in the interpretation of the results.

Numerous studies have shown the positive effects of AT in patients with FMS, specifically in reducing pain and improving overall quality of life. However, further research is ongoing to determine its effectiveness, and some studies suggest that combining AT with other treatments may be beneficial. Notably, AT may not be effective for all patients, and individual outcomes may vary. Thus, further research is needed to identify the specific AT points and optimal treatment duration for effectively managing FMS.

## Conclusion

AT currently shows some benefits in treating FMS; however, this evidence is largely based on poor-quality research. Further studies are needed to confirm these findings. Future studies should focus on original and standardized research methods and outcomes to strengthen the evidence and provide a robust support for the use of AT in the treatment of FMS.

## Data Availability

The datasets presented in this study can be found in online repositories. The names of the repository/repositories and accession number(s) can be found in the article/Supplementary material.
