# Supplementary material for: Acupuncture for fibromyalgia syndrome: an overview of systematic reviews and meta-analyses
Source: Front Med (Lausanne). 2025 Dec 5;12:1712065. doi: 10.3389/fmed.2025.1712065 (PMC12714891; doi:10.3389/fmed.2025.1712065)
Supplement: Supplementary file 1 [file Table_1.docx]

**Supplement 1. Search strategy**

**1. PubMed**

#1 fibromyalgia [MeSH]

#2 fibromyal* [ti,ab]

#3 fibromyalgia syndrome [ti,ab]

#4 chronic widespread pain [ti,ab]

#5 #1 OR #2 OR #3 OR #4

#6 acupuncture [MeSH]

#7 acupuncture therapy [MeSH]

#8 acupuncture points [MeSH]

#9 body acupuncture [ti,ab]

#10 electroacupuncture [MeSH]

#11 acupuncture [ti,ab]

#12 electro-acupuncture [ti,ab]

#13 acupuncture, ear [MeSH]

#14 auricular acupuncture [ti,ab]

#15 scalp acupuncture [ti,ab]

#16 dry needling [ti,ab]

#17 #7 OR #8 OR #9 OR #10 OR #11 OR #12 OR #13 OR #14 OR #15 OR #16

#18 systematic review [pt] OR systematic review [ti,ab] OR meta-analysis [ti,ab]

#19 #5 AND #17 AND #18

**2. Cochrane library**

#1 MeSH descriptor fibromyalgia explode all trees

#2 (“fibromyal*”):ti,ab,kw

#3 (“fibromyalgia syndrome”):ti,ab,kw

#4 (“chronic widespread pain”):ti,ab,kw

#5 (#1 OR #2 OR #3 OR #4)

#6 MeSH descriptor Acupuncture explode all trees

#7 MeSH descriptor Acupuncture Therapy explode all trees

#8 MeSH descriptor Electroacupuncture explode all trees

#9 (“acupuncture”):ti,ab,kw

#10 (“body acupuncture”):ti,ab,kw

#11 (“electro-acupuncture”):ti,ab,kw

#12 MeSH descriptor Acupuncture, Ear explode all trees

#13 (“auricular acupuncture”):ti,ab,kw

#14 (“scalp acupuncture”):ti,ab,kw

#15 (“dry needling”):ti,ab,kw

#16 (#7 OR #8 OR #9 OR #10 OR #11 OR #12 OR #13 OR #14 OR #15

#17 systematic review [pt] OR systematic review [ti,ab,kw]

#18 #5 AND #16 AND #17

**3. Embase**

#1 'fibromyalgia'/exp OR fibromyal*:ti,ab,kw OR 'fibromyalgia syndrome':ti,ab,kw OR 'chronic widespread pain':ti,ab,kw

#2 'acupuncture therapy'/exp OR 'acupuncture points'/exp OR 'electroacupuncture'/exp OR 'acupuncture, ear'/exp OR 'acupuncture'/exp OR 'body acupuncture':ti,ab,kw OR 'electro acupuncture':ti,ab,kw OR 'auricular acupuncture':ti,ab,kw OR 'scalp acupuncture':ti,ab,kw OR 'dry needling':ti,ab,kw OR 'trigger point':ti,ab,kw OR 'acupoint injection':ti,ab,kw

#3 'systematic review':ti,ab,kw

#4 #1 AND #2 AND #3

**4. CNKI and Wanfang**

1 纤维肌痛

2 纤维肌痛综合征

3 1 OR 2

4 针

5 针刺

6 针灸

7 电针

8 耳针

9 头针

10 4 OR 5 OR 6 OR 7 OR 8 OR 9 OR

11 Meta分析

12 [系统评价](https://oversea.cnki.net/kns/Detail?sfield=fn&QueryID=13&CurRec=13&DbCode=CJFD&dbname=CJFDLAST2017&filename=HNZB201705035)

13. 11 OR 12

14. 3 AND 10 AND 13

**7. RISS and KISS and OASIS and KTKP (한국전통지식포탈)**

섬유근육통 AND 침

fibromyalgia AND acupuncture

**10. KMbase**

섬유근육통 (논문제목, 키워드, 초록) AND 침 (논문제목, 키워드, 초록) AND 체계적문헌고찰 (논문제목, 키워드, 초록)

fibromyalgia(논문제목, 키워드, 초록) AND acupuncture(논문제목, 키워드, 초록) AND systematic review (논문제목, 키워드, 초록)

**11. KoreaMed**

1. fibromyalgia [MH]

2. fibromyalgia [TIAB]

3. fibromyalgia syndrome [TIAB]

4. chronic widespread pain [TIAB]

5. 1 OR 2 OR 3 OR 4

6 acupuncture [MH]

7 acupuncture therapy [MH]

8 acupuncture points [MH]

9 body acupuncture [TIAB]

10 electroacupuncture [MH]

11 electro-acupuncture [TIAB]

12 electro-acupuncture [TIAB]

13 auricular acupuncture [TIAB]

14 scalp acupuncture [TIAB]

15 dry needling [TIAB]

16 6 OR 7 OR 8 OR 9 OR 10 OR 11 OR 12 OR 13 OR 14 OR 15

17 systematic review [TIAB] OR meta-analysis [TIAB]

18. 5 AND 16 AND 17

**Supplement 2. Relationships between systematic reviews and primary studies.**

|  |  |  | **13 Systematic review** | | | | | | | | | | | | |
| --- | --- | --- | --- | --- | --- | --- | --- | --- | --- | --- | --- | --- | --- | --- | --- |
|  |  |  | Mayhew  2007 ^1^ | Daya  2007 ^2^ | Martin-Sanchez  2009 ^3^ | Langhorst  2010 ^4^ | Gao  2010 ^5^ | Cao  2013 ^6^ | Deare  2013 ^7^ | Bai  2014 ^8^ | Kim  2019 ^9^ | Zhang  2019 ^10^ | Sarmiento-Hernandez  2020 ^11^ | Zheng  2022 ^12^ | Yang  2022 ^13^ |
|  |  |  | 5 RCT | 4 RCT | 6 RCT | 7 RCT | 5 RCT | 16 RCT | 9 RCT | 9 RCT | 10 RCT | 12 RCT | 13 RCT | 13 RCT | 14 RCTs |
| **45 RCT** | Lautensclauger 1989 ^14^ | 3 |  |  |  |  |  |  |  |  |  |  |  |  |  |
|  | Deluze 1992 ^15^ | 9 |  |  |  |  |  |  |  |  |  |  |  |  |  |
|  | Sprott 1998 ^16^ | 6 |  |  |  |  |  |  |  |  |  |  |  |  |  |
|  | Sandberg 1999 ^17^ | 1 |  |  |  |  |  |  |  |  |  |  |  |  |  |
|  | Zhang 2001 ^18^ | 2 |  |  |  |  |  |  |  |  |  |  |  |  |  |
|  | Wang 2002 ^19^ | 3 |  |  |  |  |  |  |  |  |  |  |  |  |  |
|  | Liu 2002 ^20^ | 3 |  |  |  |  |  |  |  |  |  |  |  |  |  |
|  | Cao 2003 ^21^ | 1 |  |  |  |  |  |  |  |  |  |  |  |  |  |
|  | Guo 2003 ^22^ | 2 |  |  |  |  |  |  |  |  |  |  |  |  |  |
|  | Guo 2004 ^23^ | 1 |  |  |  |  |  |  |  |  |  |  |  |  |  |
|  | Wang 2004 ^24^ | 1 |  |  |  |  |  |  |  |  |  |  |  |  |  |
|  | Guo 2005 ^25^ | 2 |  |  |  |  |  |  |  |  |  |  |  |  |  |
|  | Guo 2005 b ^26^ | 4 |  |  |  |  |  |  |  |  |  |  |  |  |  |
|  | Assefi 2005 ^27^ | 10 |  |  |  |  |  |  |  |  |  |  |  |  |  |
|  | Harris 2005 ^28^ | 9 |  |  |  |  |  |  |  |  |  |  |  |  |  |
|  | Sun 2005 ^29^ | 1 |  |  |  |  |  |  |  |  |  |  |  |  |  |
|  | Martin 2006 ^30^ | 11 |  |  |  |  |  |  |  |  |  |  |  |  |  |
|  | Singh 2006 ^31^ | 2 |  |  |  |  |  |  |  |  |  |  |  |  |  |
|  | Yao 2006 ^32^ | 2 |  |  |  |  |  |  |  |  |  |  |  |  |  |
|  | Targino 2008 ^33^ | 3 |  |  |  |  |  |  |  |  |  |  |  |  |  |
|  | Wang 2008 ^34^ | 1 |  |  |  |  |  |  |  |  |  |  |  |  |  |
|  | Harris 2008 ^35^ | 1 |  |  |  |  |  |  |  |  |  |  |  |  |  |
|  | Harris 2009 ^36^ | 7 |  |  |  |  |  |  |  |  |  |  |  |  |  |
|  | Gong 2010 ^37^ | 3 |  |  |  |  |  |  |  |  |  |  |  |  |  |
|  | Guo 2010 ^38^ | 1 |  |  |  |  |  |  |  |  |  |  |  |  |  |
|  | Itoh 2010 ^39^ | 1 |  |  |  |  |  |  |  |  |  |  |  |  |  |
|  | Jang 2010 ^40^ | 1 |  |  |  |  |  |  |  |  |  |  |  |  |  |
|  | Ruan 2010 ^41^ | 1 |  |  |  |  |  |  |  |  |  |  |  |  |  |
|  | Hadianfard 2012 ^42^ | 2 |  |  |  |  |  |  |  |  |  |  |  |  |  |
|  | Liu 2012 ^43^ | 2 |  |  |  |  |  |  |  |  |  |  |  |  |  |
|  | Tabatabaee 2012 ^44^ | 1 |  |  |  |  |  |  |  |  |  |  |  |  |  |
|  | Casanueva 2013 ^45^ | 1 |  |  |  |  |  |  |  |  |  |  |  |  |  |
|  | Harte 2013 ^46^ | 2 |  |  |  |  |  |  |  |  |  |  |  |  |  |
|  | Zhao 2013 ^47^ | 1 |  |  |  |  |  |  |  |  |  |  |  |  |  |
|  | Stival 2014 ^48^ | 4 |  |  |  |  |  |  |  |  |  |  |  |  |  |
|  | Araujo 2016 ^49^ | 1 |  |  |  |  |  |  |  |  |  |  |  |  |  |
|  | Vas 2016 ^50^ | 3 |  |  |  |  |  |  |  |  |  |  |  |  |  |
|  | Castro 2017 ^51^ | 1 |  |  |  |  |  |  |  |  |  |  |  |  |  |
|  | Uğurlu 2017 ^52^ | 4 |  |  |  |  |  |  |  |  |  |  |  |  |  |
|  | Zucker 2017 ^53^ | 1 |  |  |  |  |  |  |  |  |  |  |  |  |  |
|  | Castro 2018 ^54^ | 1 |  |  |  |  |  |  |  |  |  |  |  |  |  |
|  | Karatay 2018 ^55^ | 4 |  |  |  |  |  |  |  |  |  |  |  |  |  |
|  | Mampel 2018 ^56^ | 1 |  |  |  |  |  |  |  |  |  |  |  |  |  |
|  | Ye 2019 ^57^ | 1 |  |  |  |  |  |  |  |  |  |  |  |  |  |
|  | Mawla 2021 ^58^ | 1 |  |  |  |  |  |  |  |  |  |  |  |  |  |

RCT: randomised contrilles trilas

**References**

1. Mayhew E, Ernst E. Acupuncture for fibromyalgia-a systematic review of randomized clinical trials. 1. *Rheumatology (Oxford)*. May 2007;46(5):801-4. doi:10.1093/rheumatology/kel406

2. Daya S. The efficacy of acupuncture in the treatment of fibromyalgia syndrome. 2. *Journal of the acupuncture association of chartered physiotherapists*. 2007;(3):35‐46.

3. Martin-Sanchez E, Torralba E, Díaz-Domínguez E, Barriga A, Martin JL. Efficacy of acupuncture for the treatment of fibromyalgia: systematic review and meta-analysis of randomized trials. 3. *Open Rheumatol J*. Jun 16 2009;3:25-9. doi:10.2174/1874312900903010025

4. Langhorst J, Klose P, Musial F, Irnich D, Häuser W. Efficacy of acupuncture in fibromyalgia syndrome--a systematic review with a meta-analysis of controlled clinical trials. 4. *Rheumatology (Oxford)*. Apr 2010;49(4):778-88. doi:10.1093/rheumatology/kep439

5. Gao X, Du YH, Li B, et al. Comparing the effectiveness of acupuncture and western medicine in the treatment of fibromyalgia syndrome: A systematic review. 5. *Chinese Journal of Pain Medicine*. 2010;16(2):112-114.

6. Cao H, Li X, Han M, Liu J. Acupoint stimulation for fibromyalgia: a systematic review of randomized controlled trials. 6. *Evid Based Complement Alternat Med*. 2013;2013:362831. doi:10.1155/2013/362831

7. Deare JC, Zheng Z, Xue CC, et al. Acupuncture for treating fibromyalgia. 7. *Cochrane Database Syst Rev*. May 31 2013;2013(5):Cd007070. doi:10.1002/14651858.CD007070.pub2

8. Yang B, Yi G, Hong W, et al. Efficacy of acupuncture on fibromyalgia syndrome: a meta-analysis. 8. *J Tradit Chin Med*. Aug 2014;34(4):381-91. doi:10.1016/s0254-6272(15)30037-6

9. Kim J, Kim SR, Lee H, Nam DH. Comparing verum and sham acupuncture in fibromyalgia syndrome: A systematic review and meta-analysis. 9. *Evid Based Complement Alternat Med*. 2019;2019:8757685. doi:10.1155/2019/8757685

10. Zhang XC, Chen H, Xu WT, Song YY, Gu YH, Ni GX. Acupuncture therapy for fibromyalgia: a systematic review and meta-analysis of randomized controlled trials. 10. *J Pain Res*. 2019;12:527-542. doi:10.2147/jpr.S186227

11. Sarmiento-Hernández I, Pérez-Marín M, Nunez-Nagy S, Pecos-Martín D, Gallego-Izquierdo T, Sosa-Reina MD. Effectiveness of invasive techniques in patients with fibromyalgia: Systematic review and meta-analysis. 11. *Pain Med*. Dec 25 2020;21(12):3499-3511. doi:10.1093/pm/pnaa321

12. Zheng C, Zhou T. Effect of acupuncture on pain, fatigue, sleep, physical function, stiffness, Well-Being, and Safety in Fibromyalgia: A Systematic Review and Meta-Analysis. 12. *J Pain Res*. 2022;15:315-329. doi:10.2147/jpr.S351320

13. Yang P, Su SY, Wang T, Li L. Meta-analysis of the effectiveness of acupuncture in the treatment of fibromyalgia. 13. *Chinese Journal of Pain Medicine*. 2022;28(12):944-950.

14. Lautenschlager J, Schnorrenberger CC, Muller W. Acupuncture in the treatment of generalized tendomyopathy (fibromyalgia syndrome). R1. *Deutsche Zeitschrift fur Akupunktur [German Journal of Acupuncture and Related Techniques]*. 1989;32:122-128.

15. Deluze C, Bosia L, Zirbs A, Chantraine A, Vischer TL. Electroacupuncture in fibromyalgia: results of a controlled trial. R2. *Bmj*. Nov 21 1992;305(6864):1249-1252. doi:10.1136/bmj.305.6864.1249

16. Sprott H. Efficiency of acupuncture in patients with fibromyalgia. R3. *Clinical Bulletin of Myofascial Therapy*. 1998;3(1):37-43.

17. Sandberg M, Lundeberg T, Gerdle B. Manual acupuncture in fibromyalgia: a long-term pilot study. R4. *J Musculoskelet Pain*. 1999;7(3):39-58.

18. Zhang YG. Clinical observatuion on acupuncture treatment of primary fibromyalgia syndrome. R5. *Zhong Guo Zhen Jiu* 2001;21(1):19-20.

19. Wang SP, Wang XF, Zhang DX, Xia P, Yang HX. Clinical observation on therapeutic effect of acupuncture treatment based on syndrome differentiation of meridians on fibromyalgia syndrome. R6. *Zhong Guo Zhen Jiu*. 2002;22(12):807-809.

20. Liu Q, Li F. Clinical observation of acupuncture for 30 cases of fibromyalgia. R7. *Anthology of Medicine*. 2002;21(2):183-184.

21. Cao Q, Li Y. Combination of acupuncture and antide pressant medications in treating of 56 cases of fibromyalgia. R8. *Chinese Archives of Traditional Chinese Medicine*. 2003;21(5):813-817.

22. Guo XJ, Jia J. Comparison of therapeutic effects of transcutaneous electrical nerve stimulation and electroacupuncture on fibromyolgia syndrome. R9. *Zhong Guo Zhen Jiu* 2003;(11):24-26.

23. Guo XJ, Jia J. Comparison of therapeutic effects on fibromyalgia syndrome between dermal-neurological electric stimulation and electric acupuncture. R10. *Zhong Hua Wu Li Yi Xue Yu Kang Fu Za Zhi*. 2004;26(2):582-588.

24. Wang WX, Liu ZT, Wu YC, Wu X. Clinical observation on 42 cases of fibromyalgia syndrome treated by acupuncture and moxibustion syndrome differentiation. R11. *Forum On Traditional Chinese Medicine*. 2004;19(1):26-27.

25. Guo Y, Sun YZ. Clinical study on treatment of fibromyal gia syndrome with penetration needling at the back. R12. *Zhong Guo Zhen Jiu*. 2005;25(2):98-100.

26. Guo Y SY. Clinical study on treatment of fibromyalgia syndrome with penetration needling at the back. R13. *Zhong Guo Zhen Jiu* 2005;25(2):98-100.

27. Assefi NP, Sherman KJ, Jacobsen C, Goldberg J, Smith WR, Buchwald D. A randomized clinical trial of acupuncture compared with sham acupuncture in fibromyalgia. R14. *Ann Intern Med*. Jul 5 2005;143(1):10-9. doi:10.7326/0003-4819-143-1-200507050-00005

28. Harris RE, Tian X, Williams DA, et al. Treatment of fibromyalgia with formula acupuncture: investigation of needle placement, needle stimulation, and treatment frequency. R15. *J Altern Complement Med*. Aug 2005;11(4):663-671. doi:10.1089/acm.2005.11.663

29. Sun YZ, Guo Y, Zhang L. Clinical study on back acupoint therapy in the treatment of primary fibromyalgia syndrome. R16. *International Symposium on Acupuncture Techniques and Clinical Applications*. 2005;国际针灸技法及临床应用学术研讨会 . 北京

30. Martin DP, Sletten CD, Williams BA, Berger IH. Improvement in fibromyalgia symptoms with acupuncture: results of a randomized controlled trial. R17. *Mayo Clin Proc*. Jun 2006;81(6):749-757. doi:10.4065/81.6.749

31. Singh BB, Wu WS, Hwang SH, et al. Effectiveness of acupuncture in the treatment of fibromyalgia. R18. *Altern Ther Health Med*. Mar-Apr 2006;12(2):34-41.

32. Yao QP. Clinical study on fibromyalgia syndrome with the acupuncture of stimulating Du channel and smoothing other channels and collaterals R19. *Heilongjiang University of Traditional Chinese Medicine*. 2006;

33. Targino RA, Imamura M, Kaziyama HH, et al. A randomized controlled trial of acupuncture added to usual treatment for fibromyalgia. R20. *J Rehabil Med*. Jul 2008;40(7):582-588. doi:10.2340/16501977-0216

34. Wang CM. Clinical treatment of acupuncture and semiconductor laser irradiation on fibromyalgia symptoms 28 cases. R21. *Shi Yong Zhong Yi Nei Ke Xue Za Zhi*. 2008;22(1):58.

35. Harris RE, Sundgren PC, Pang Y, et al. Dynamic levels of glutamate within the insula are associated with improvements in multiple pain domains in fibromyalgia. R22. *Arthritis Rheum*. Mar 2008;58(3):903-907. doi:10.1002/art.23223

36. Harris RE, Zubieta JK, Scott DJ, Napadow V, Gracely RH, Clauw DJ. Traditional Chinese acupuncture and placebo (sham) acupuncture are differentiated by their effects on mu-opioid receptors (MORs). R23. *Neuroimage*. Sep 2009;47(3):1077-1085. doi:10.1016/j.neuroimage.2009.05.083

37. Gong WZ, Wang YQ. Observations on the therapeutic effect of acupuncture on fibromyalgia syndrom. R24. *Shanghai J Acup Moxi* 2010;29(11):725-727.

38. Gou AS, Li AH. Clinical observation on therapeutic effect of elecacupuncture combining TDP irradiation on fibromyalgia syndrome 36 cases. R25. *Jiao Tong Yi Xue*. 2010;24(4):410-411.

39. Itoh K, Kitakoji H. Effects of acupuncture to treat fibromyalgia: a preliminary randomised controlled trial. R26. *Chin Med*. Mar 23 2010;5:11. doi:10.1186/1749-8546-5-11

40. Jang ZY, Li CD, Qiu L, et al. [Combination of acupuncture, cupping and medicine for treatment of fibromyalgia syndrome: a multi-central randomized controlled trial]. R27. *Zhong Guo Zhen Jiu* Apr 2010;30(4):265-269.

41. Ruan YD, Wei WZ, Hong XT, Yang XH. Clinical observation of moxibustion for fibromyalgia. R28. *11th Acupuncture Symposium of Guangdong Province*. 2010;

42. Hadianfard MJ, Hosseinzadeh Parizi M. A randomized clinical trial of fibromyalgia treatment with acupuncture compared with fluoxetine. R29. *Iran Red Crescent Med J*. Oct 2012;14(10):631-40.

43. Liu ZJ. Clinical study on treating fibromyalgia syndrome with acupuncture and point injection R30. *Hubei University Chinese Medicine [Thesis]*. 2012;

44. Tabatabaee M. Comparison of acupuncture and fluoxetine in improving of fibromyalgia symptoms. R31. *Nanjing University of Traditional Chinese Medicine [Thesis]*. 2012;

45. Casanueva B, Rivas P, Rodero B, Quintial C, Llorca J, González-Gay MA. Short-term improvement following dry needle stimulation of tender points in fibromyalgia. R32. *Rheumatol Int*. Jun 2014;34(6):861-6. doi:10.1007/s00296-013-2759-3

46. Harte SE, Clauw DJ, Napadow V, Harris RE. Pressure pain sensitivity and insular combined glutamate and glutamine (Glx) are associated with subsequent clinical response to sham but not traditional acupuncture in patients who have chronic pain. R33. *Med Acupunct*. Apr 2013;25(2):154-160. doi:10.1089/acu.2013.0965

47. Shao ML, Jiang M. Acupuncture for soothing the liver and relieving depression in the treatment of fibromyalgia syndrome. R34. *Hubei J Tradit Chin Med*. 2013;35(12):62.

48. Stival RS, Cavalheiro PR, Stasiak CE, Galdino DT, Hoekstra BE, Schafranski MD. [Acupuncture in fibromyalgia: a randomized, controlled study addressing the immediate pain response]. R35. *Rev Bras Reumatol*. Nov-Dec 2014;54(6):431-6. Acupuntura na fibromialgia: um estudo randomizado-controlado abordando a resposta imediata da dor. doi:10.1016/j.rbr.2014.06.001

49. Dias PA, Guimarães AB, Albuquerque Ade O, de Oliveira KL, Cavalcante ML, Guimarães SB. Short-term complementary and alternative medicine on quality of life in women with fibromyalgia. R36. *J Integr Med*. Jan 2016;14(1):29-35. doi:10.1016/s2095-4964(16)60235-2

50. Vas J, Santos-Rey K, Navarro-Pablo R, et al. Acupuncture for fibromyalgia in primary care: a randomised controlled trial. R37. *Acupunct Med*. Aug 2016;34(4):257-66. doi:10.1136/acupmed-2015-010950

51. Castro-Sanchez AM, Garcia-Lopez H, Mataran-Penarrocha GA, et al. Effects of dry needling on spinal mobility and trigger points in patients with fibromyalgia syndrome. R38. *Pain Physician*. Feb 2017;20(2):37-52.

52. Uğurlu FG, Sezer N, Aktekin L, Fidan F, Tok F, Akkuş S. The effects of acupuncture versus sham acupuncture in the treatment of fibromyalgia: a randomized controlled clinical trial. R39. *Acta Reumatol Port*. Jan-Mar 2017;42(1):32-37. The effects of acupuncture versus sham acupuncture in the treatment of fibromyalgia: a randomized controlled clinical trial.

53. Zucker NA, Tsodikov A, Mist SD, Cina S, Napadow V, Harris RE. Evoked pressure pain sensitivity is associated with differential analgesic response to verum and sham acupuncture in fibromyalgia. R40. *Pain Med*. Aug 1 2017;18(8):1582-1592. doi:10.1093/pm/pnx001

54. Castro Sánchez AM, García López H, Fernández Sánchez M, et al. Improvement in clinical outcomes after dry needling versus myofascial release on pain pressure thresholds, quality of life, fatigue, pain intensity, quality of sleep, anxiety, and depression in patients with fibromyalgia syndrome. R41. *Disabil Rehabil*. Sep 2019;41(19):2235-2246. doi:10.1080/09638288.2018.1461259

55. Karatay S, Okur SC, Uzkeser H, Yildirim K, Akcay F. Effects of acupuncture treatment on fibromyalgia symptoms, serotonin, and substance P levels: A randomized sham and placebo-controlled clinical trial. R42. *Pain Med*. Mar 1 2018;19(3):615-628. doi:10.1093/pm/pnx263

56. Mampel JV. Eficacia analgesica del tratamiento invasivo miofascial (puncion seca) en fibromialgia. R43. *Agora Salut* 2018;5(13):119-128.

57. Ye YR, Wang J. Effect of acupuncture on the sleep and life quality in the fibromyalgia syndrome patients. R44. *World Journal of Sleep Medicine*. 2019;6(8):1054-1056.

58. Mawla I, Ichesco E, Zöllner HJ, et al. Greater somatosensory afference with acupuncture increases primary somatosensory connectivity and alleviates fibromyalgia pain via insular γ-aminobutyric acid: A randomized neuroimaging trial. R45. *Arthritis Rheumatol*. Jul 2021;73(7):1318-1328. doi:10.1002/art.41620

**Supplement 3. Outcomes used in SRs/MAs**

| **Pain intensity and relief** | **Frequency** |
| --- | --- |
| Pain (Mix) | 5 |
| Pain VAS | 6 |
| PPT | 2 |
| ER | 3 |
| Pain NRS | 1 |
| Pain SF-MPQ | 1 |
| Changes of pain VAS | 1 |
| No. of tender points | 2 |
| **Comorbidities** |  |
| Fatigue | 5 |
| Sleep | 5 |
| Depression | 2 |
| Stiffness | 2 |
| **Quality of life** |  |
| FIQ | 6 |
| Quality of life (SF-36) | 4 |
| Well-being | 2 |

ER: efficacy rate; FIQ: fibromyalgia impact questionnaire; NRS: numerical rating scale; PPT: pain pressure threshold; VAS: visual analogue scale; QOL: Quality of life

**Supplement 4. Quality of evidence in the included SRs assessed by the GRADE approach**

| First author (year) | Intervention vs. comparator | Outcome | No. of Studies  (No. of Patients) | Effect Size SMD  (95% CI) | P-value | Heterogeneity I^2^ | Risk of bias | Inconsistency | Indirectness | Imprecision | Publication bias | Quality of  evidence |
| --- | --- | --- | --- | --- | --- | --- | --- | --- | --- | --- | --- | --- |
| Martin-Sanchez (2009) | AT vs. Sham AT | Pain VAS | 4 (257) | SMD 0.02 [-0.24, 0.28] | 0.87 | 0 | Serious^①^ | None | None | None | None | Moderate |
| Langhorst (2010) | AT vs. Sham AT | Pain | 7 (242) | SMD -0.25 [-0.49, -0.02] | 0.04 | 1 | Serious^①^ | None | None | None | None | Moderate |
|  |  | Fatigue | 3 (147) | SMD 0.04 [-0.32, 0.39] | 0.84 | 16 | Serious^①^ | None | Serious^③^ | None | Serious^④^ | Very low |
|  |  | Sleep | 2 (87) | SMD 0.05 [-0.79, 0.83] | 0.91 | 76 | Serious^①^ | Serious^③^ | Serious^③^ | None | Serious^④^ | Very low |
|  |  | Physical function (SF-36) | 3 (149) | SMD -0.15 [-0.61, 0.91] | 0.52 | 51 | Serious^①^ | None | Serious^③^ | None | Serious^④^ | Very low |
|  |  | Pain (F/U) | 2 (87) | SMD -0.11 [-0.72, 0.49] | 0.71 | 50 | Serious^①^ | None | Serious^③^ | None | Serious^④^ | Very low |
|  |  | Physical function (F/U) | 2 (87) | SMD -0.05 [-0.47, 0.37] | 0.83 | 0 | Serious^①^ | None | Serious^③^ | None | Serious^④^ | Very low |
| Gao (2010) | AT vs. Medicine | ER | 5 (190) | RR 1.49 [1.20, 1.73] | <0.00001 | 0 | Serious^①^ | None | Serious^③^ | None | Serious^④^ | Very low |
| Cao (2013) | AT vs. Sham AT | Changes of pain VAS | 6 (330) | SMD -0.09 [-0.32, 0.14] | 0.44 | 2 | Serious^①^ | None | None | None | None | Moderate |
|  |  | Pain VAS | 6 (360) | SMD -0.22 [-0.51, 0.07] | 0.13 | 26 | Serious^①^ | None | None | None | None | Moderate |
|  |  | Depression | 1 (50) | MD -0.33[-0.90, 023] | 0.25 | NA | Serious^①^ | None | Serious^③^ | None | Serious^④^ | Very low |
|  |  | FIQ | 1 (50) | MD -4.30 [-11.08, 2.48] | 0.21 | NA | Serious^①^ | None | Serious^③^ | None | Serious^④^ | Very low |
|  |  | Fatigue | 2 (164) | SMD -0.05 [-0.41, 0.30] | 0.77 | 0 | Serious^①^ | None | Serious^③^ | None | Serious^④^ | Very low |
|  | AT vs. Medicine | Pain VAS | 5 (285) | SMD -0.74 [-1.13, -0.35] | 0.0002 | 55 | Serious^①^ | None | None | None | None | Moderate |
|  |  | No. of tender points | 3 (195) | SMD -2.38 [-3.40, -1.37] | <0.00001 | 0 | Serious^①^ | None | Serious^③^ | None | Serious^④^ | Very low |
|  |  | Depression | 2 (90) | SMD -0.67 [-1.10, -0.25] | 0.02 | 0 | Serious^①^ | None | Serious^③^ | None | Serious^④^ | Very low |
|  |  | Sleep | 3 (195) | SMD -0.32 [-0.63, -0.01] | 0.04 | 0 | Serious^①^ | None | Serious^③^ | None | Serious^④^ | Very low |
|  |  | FIQ | 1 (30) | MD -4.60 [-12.42. 3.22] | 0.25 | NA | Serious^①^ | None | Serious^③^ | None | Serious^④^ | Very low |
|  |  | Fatigue | 1 (30) | MD -0.27 [-0.99, 0.45] | 0.46 | NA | Serious^①^ | None | Serious^③^ | None | Serious^④^ | Very low |
| Bai (2014) | AT vs. Sham AT | Pain NRS | 2 (105) | MD -1.06 [-10.41, 8.30] | 0.82 | 0 | Serious^①^ | None | Serious^③^ | None | Serious^④^ | Very low |
|  | AT vs. Medicine | Pain VAS | 1 (56) | MD -2.27 [-3.05, -1.49] | <0.00001 | NA | Serious^①^ | None | Serious^③^ | None | Serious^④^ | Very low |
|  |  | ER | 1 (36) | RR 1.38 [1.00, 1.91] | 0.05 | NA | Serious^①^ | None | Serious^③^ | None | Serious^④^ | Very low |
|  |  | PPT | 1 (58) | MD 0.69 [0.38, 1.00] | <0.00001 | NA | Serious^①^ | None | Serious^③^ | None | Serious^④^ | Very low |
| Deare (2013) | AT vs. Sham AT | Pain | 6 (286) | SMD -0.14 [-0.53, 0.25] | 0.48 | 54 | Serious^①^ | None | None | None | None | Moderate |
|  |  | Physical function (SF-36) | 1 (56) | MD -5.80 [-10.91, -0.69] | 0.03 | NA | Serious^①^ | None | Serious^③^ | None | Serious^④^ | Very low |
|  |  | Well-being | 3 (200) | SMD 0.29 [-0.44, 1.01] | 0.44 | 82 | Serious^①^ | Serious^③^ | None | None | None | Low |
|  |  | Sleep | 3 (200) | SMD 0.16 [-0.29, 0.61] | 0.49 | 56 | Serious^①^ | None | None | None | None | Moderate |
|  |  | Fatigue | 3 (200) | SMD -0.10 [-0.81, 0.61] | 0.78 | 81 | Serious^①^ | Serious^③^ | None | None | None | Low |
|  |  | Stiffness | 2 (104) | SMD -0.45 [-0.84, -0.06] | 0.02 | 0 | Serious^①^ | None | Serious^③^ | None | Serious^④^ | Very low |
|  |  | Pain (F/U) | 2 (145) | SMD -0.12 [-0.52, 0.28] | 0.55 | 20 | Serious^①^ | None | Serious^③^ | None | Serious^④^ | Very low |
|  |  | Well-being (F/U) | 2 (104) | SMD -0.03 [-0.87, 0.81] | 0.94 | 81 | Serious^①^ | Serious^③^ | Serious^③^ | None | Serious^④^ | Very low |
|  |  | Sleep (F/U) | 2 (104) | SMD -0.09 [-0.44, 0.26] | 0.62 | 0 | Serious^①^ | None | Serious^③^ | None | Serious^④^ | Very low |
|  |  | Fatigue (F/U) | 2 (154) | SMD 0.04 [-0.52, 0.59] | 0.9 | 57 | Serious^①^ | None | Serious^③^ | None | Serious^④^ | Very low |
|  |  | Stiffness (F/U) | 1 (49) | MD -0.30 [-1.60, 1.00] | 0.35 | NA | Serious^①^ | None | Serious^③^ | None | Serious^④^ | Very low |
| Kim (2019) | AT vs. Sham AT | Pain | 11 (559) | SMD -0.49 [-0.79, -0.20] | 0.001 | 59 | Serious^①^ | None | None | None | None | Moderate |
|  |  | FIQ | 6 (389) | SMD -0.69 [-0.91, -0.47] | <0.00001 | 4 | Serious^①^ | None | None | None | None | Moderate |
| Zhang (2019) | AT vs. Sham AT | Pain VAS | 9 (528) | MD -1.04 [-1.70, -0.38] | 0.002 | 78 | Serious^①^ | Serious^③^ | None | None | None | Low |
|  |  | Pain SF-MPQ | 2 (70) | MD -1.23 [-4.74, 2.27] | 0.49 | 0 | Serious^①^ | None | Serious^③^ | None | Serious^④^ | Low |
|  |  | FIQ | 5 (307) | MD -13.39 [-21.69, -5.10] | 0.002 | 82 | Serious^①^ | Serious^③^ | None | None | None | Moderate |
|  |  | Pain (F/U) | 3 (251) | MD -1.58 [-2.72, -0.44] | 0.006 | 67 | Serious^①^ | None | None | None | None | Moderate |
|  |  | FIQ (F/U) | 3 (251) | MD -12.92 [-24.92, -0.93] | 0.03 | 81 | Serious^①^ | Serious^③^ | None | None | None | Low |
|  | AT vs. Medicine | Pain VAS | 2 (98) | MD -1.81 [-2.43, -1.18] | <0.00001 | 0 | Serious^①^ | None | Serious^③^ | None | Serious^④^ | Very low |
|  |  | Pain VAS (F/U) | 1 (60) | MD -2.11 [-2.97, -1.25] | <0.00001 | NA | Serious^①^ | None | Serious^③^ | None | Serious^④^ | Very low |
| Isabel (2020) | AT vs. Control (Sham AT, UC) | Pain | 7 (372) | SMD -0.94 [- 1.44, -0.44] | 0.0002 | 79 | Serious^①^ | Serious^③^ | None | None | None | Low |
|  |  | FIQ | 5 (310) | SMD -0.99 [-1.69, 0.29] | 0.006 | 87 | Serious^①^ | Serious^③^ | None | None | None | Low |
|  |  | PPT | 2 (184) | SMD 0.31 [0.02, 0.61] | 0.04 | 0 | Serious^①^ | None | Serious^③^ | None | Serious^④^ | Very low |
|  |  | QOL SF-36 | 3 (211) | SMD 0.84 [0.30, 1.38] | 0.002 | 69 | Serious^①^ | None | None | None | None | Moderate |
| Zheng (2022) | AT vs. Sham AT | Pain | 12 (715) | SMD -0.42 [-0.66, -0.17] | 0.0009 | 58 | Serious^①^ | None | None | None | None | Moderate |
|  |  | Fatigue | 4 (251) | SMD -0.03 [-0.42, 0.35] | 0.87 | 54 | Serious^①^ | None | None | None | None | Moderate |
|  |  | Sleep | 2 (151) | SMD -0.38 [-0.78, 0.02] | 0.06 | 0 | Serious^①^ | None | Serious^③^ | None | Serious^④^ | Very low |
|  |  | Physical function (SF-36) | 3 (268) | SMD 0.14 [-0.51, 0.79] | 0.67 | 83 | Serious^①^ | Serious^③^ | None | None | None | Low |
|  |  | Stiffness | 2 (104) | SMD -0.38 [-0.77, 0.01] | 0.06 | 27 | Serious^①^ | None | Serious^③^ | None | Serious^④^ | Very low |
|  |  | Well-being | 4 (357) | SMD -0.86 [-1.49, -0.24] | 0.007 | 85 | Serious^①^ | Serious^③^ | None | None | None | Low |
|  |  | Pain (F/U) | 4 (356) | SMD -0.40 [-0.77, -0.03] | 0.03 | 60 | Serious^①^ | None | None | None | None | Moderate |
|  |  | Fatigue (F/U) | 2 (145) | SMD 0.04 [-0.52, 0.59] | 0.90 | 58 | Serious^①^ | None | Serious^③^ | None | Serious^④^ | Low |
|  |  | Sleep (F/U) | 2 (98) | SMD -0.64 [-1.77, 0.49] | 0.27 | 86 | Serious^①^ | Serious^③^ | Serious^③^ | None | Serious^④^ | Very low |
|  |  | Physical function (F/U) | 2 (98) | SMD -0.25 [-0.91, 0.41] | 0.46 | 63 | Serious^①^ | None | Serious^③^ | None | Serious^④^ | Very low |
|  |  | Well-being (F/U) | 3 (307) | SMD -0.58 [-0.82, -0.35] | <0.00001 | 0 | Serious^①^ | None | None | None | None | Moderate |

ER: efficacy rate; FIQ: fibromyalgia impact questionnaire; NRS: numerical rating scale; PPT: pain pressure threshold; VAS: visual analogue scale; QOL: Quality of life; RR: risk ratio; SAS: sham acupoint stimulation; SAT: sham acupuncture; SMD: standardized mean difference; WMD: weighted mean difference; UC: usual care. ① RCTs did not mention or not sue the blinding method and randomized grouping. ② Evidence of significant interstudy heterogeneity. ③ Confidence interval is not narrow enough, or the sample size is small. ④ Fewer studies are included, and there may be greater publication bias.

**Supplement 5. Adverse effects report in SRs/MAs.**

| **Adverse effects** | **Frequency** |
| --- | --- |
| Bruising | 6 |
| Soreness | 4 |
| Discomfort of needle insertion | 3 |
| Nausea | 3 |
| Vasovagal symptoms | 3 |
| Fainting | 2 |
| Aggravation of symptoms | 1 |
| Bleeding | 1 |
| Constipation | 1 |
| Dizziness | 1 |
| Headache | 1 |
| Local edema | 1 |
| Mouth dryness | 1 |
| Palpitation | 1 |
| Perspiration | 1 |
| Tiredness | 1 |
